# Dysregulation of the immune response in TGF-β signalopathies

**DOI:** 10.3389/fimmu.2022.1066375

**Published:** 2022-12-09

**Authors:** Marco M. Rodari, Nadine Cerf-Bensussan, Marianna Parlato

**Affiliations:** Université Paris-Cité, Institute Imagine, Laboratory of Intestinal Immunity, INSERM U1163, Paris, France

**Keywords:** TGF-β, immune dysregulation, Loeys-Dietz syndrome, TGF-β signalopathies, inflammatory bowel disease

## Abstract

The transforming growth factor-β (TGF-β) family of cytokines exerts pleiotropic functions during embryonic development, tissue homeostasis and repair as well as within the immune system. Single gene defects in individual component of this signaling machinery cause defined Mendelian diseases associated with aberrant activation of TGF-β signaling, ultimately leading to impaired development, immune responses or both. Gene defects that affect members of the TGF-β cytokine family result in more restricted phenotypes, while those affecting downstream components of the signaling machinery induce broader defects. These rare disorders, also known as TGF-β signalopathies, provide the unique opportunity to improve our understanding of the role and the relevance of the TGF-β signaling in the human immune system. Here, we summarize this elaborate signaling pathway, review the diverse clinical presentations and immunological phenotypes observed in these patients and discuss the phenotypic overlap between humans and mice genetically deficient for individual components of the TGF-β signaling cascade.

## Introduction

**Figure 1:**
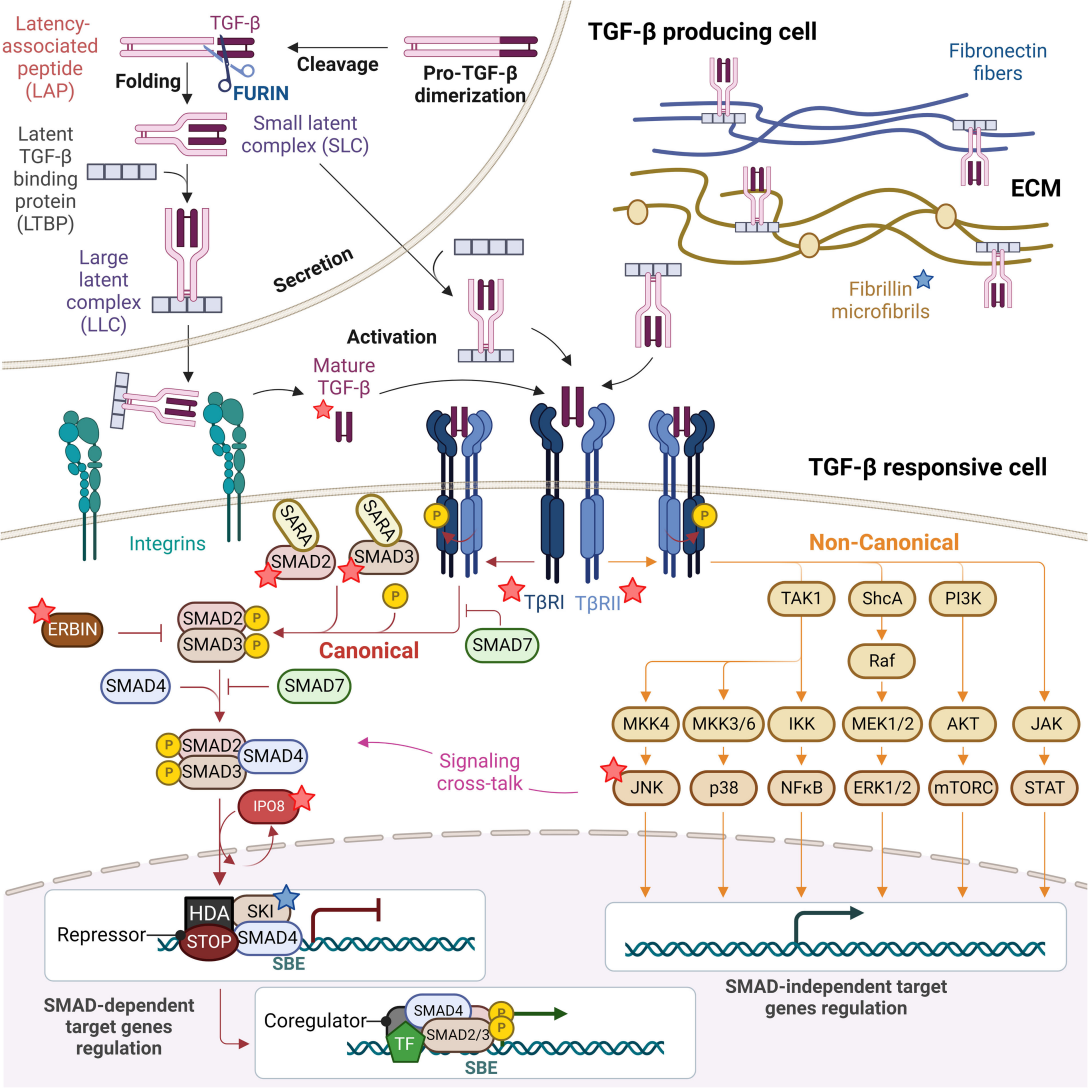
TGF-β signaling pathway: from TGF-β biogenesis to gene expression regulation. Mature TGF-β is secreted in form of small and large latent complexes (SLC and LLC) and associates with fibronectin fibers and fibrillin microfibrils composing the extracellular matrix (ECM), as well as with plasma membrane-associated proteins (not shown). Upon interaction with extracellular proteases and integrins on the surface of TGF-β responsive cells, mature TGF-β is released. Active TGF-β binds to type II receptors (TβRII), which recruits and activates type I receptors (TβRI). Receptor-induced phosphorylation of SMAD2 and SMAD3 induces their dissociation from SARA and formation of SMAD2/3-SMAD4 complexes which in turn translocate to the nucleus in an IPO8-mediated manner to initiate signaling. In the absence of TGF-β, SKI is bound to DNA at Smad Binding Elements (SBEs) with SMAD4, recruiting others repressors and histone deacetylases (HDA) to inhibit the expression of TGF-β target genes. SMAD6/7 attenuate ligand-induced SMAD activation, preventing SMAD2/3 recruitment to TβRI and competing with SMAD4 for binding to SMAD2/3, while ERBIN competes with SARA for binding to SMAD2/3, segregating phosphorylated SMAD2/3 in the cytoplasm. The TGF-β TβRI/TβRII complex can also activate non-canonical non-SMAD signaling pathways, including the ERK MAP kinase and the p38 MAPK and JNK pathways, the PI3K-AKT-mTOR and the JAK-STAT signaling, which overall complement SMAD-dependent signaling and strongly complicate the output of a TGF-β trigger. Proteins found mutated in TGF-β signalopathies are indicated with a star, blue if patients do not present with immune dysregulation (fibrillin and SKI), red if they present with immune dysregulation (TGF-β1, TGF-βRI and TGF-βRII, SMAD2 and SMAD3, IPO8, ERBIN and JNK).

The TGF-β family comprises a group of pleiotropic cytokines exerting a broad range of responses on both immune and non-immune cells. The key role of TGF-β in immunity has been established in genetically-engineered mice. This cytokine orchestrates T lymphocyte regulation by limiting effector T cell functions and promoting regulatory T cell development and functions. In humans, genome wide association studies (GWAS) have linked the SMAD3 locus - a key component of the TGF-β pathway - to predisposition to inflammatory bowel disease (IBD) and asthma (1). In addition, observations in patients carrying single gene defects in individual components of the TGF-β family (such as TGF-β1) (2) and their downstream signaling components (such as TGFβ-R1/2 or SMAD2/3) have further underscored the role of TGF-β in the pathogenesis of intestinal inflammation and allergic diseases. In addition to gene defects that directly affect the TGF-β cytokine family signaling, others affect TGF-β responses indirectly by impacting the nuclear transport of signaling components (such as IPO8) (3), or by interfering with cross-regulatory non-canonical pathways (such as ERBIN or JNK1) (4, 5). Here, we provide an overview of the TGF-β signaling pathway (**Figure 1**) and review Mendelian disorders affecting components of the TGF-β signaling machinery, also known as TGF-β signalopathies (6). These defects commonly result in a complex syndrome combining craniofacial, cardiovascular and skeletal defects, features that have been extensively reviewed elsewhere (6, 7). Here, we intend to focus on features of immune dysregulation which are also associated with these defects and often overlooked and to discuss the possible mechanisms underlying abnormal TGF-β signaling and disease pathology.

## The TGF-β signaling pathway

The human TGF-β family includes thirty-three genes that encode for homodimeric or heterodimeric secreted cytokines, including TGF-β1, TGF-β2 and TGF-β3, activins, nodals, bone morphogenetic proteins (BMPs), and growth and differentiation factors (GDFs). Identified in 1983 and best characterized (8), TGF-β1 remains the most emblematic member of the family and, as discussed below, the key actor of the family in the immune system. TGF-β1 is synthesized in the rough endoplasmic reticulum in precursor form featuring a large ~250 residue amino-signal peptide, a central pro-domain, called latency associated protein (LAP), and a short ~110 residue carboxy-terminal mature peptide. After initial biogenesis and signal peptide cleavage, two pro-peptides dimerize *via* intermolecular disulfide bonds and translocate into the Golgi complex, where TGF-β1 is cleaved from LAP by FURIN. The two products remain non-covalently bound and form the small latent complex (SLC) (9). In this conformation, the SLC is inactive as mature TGF-β is hidden and protected deep inside the protein structure (10). The SLC typically associates with either a family of proteins called latent TGF-β binding proteins (LTBPs), forming the large latent complex (LLC) that in turn associates with the extra-cellular matrix (ECM) upon secretion (11, 12), or with membrane proteins GARP (glycoprotein-A repetitions predominant) (13). Several mechanisms regulate the release of active TGF-β, including αV integrins expressed at the cell surface of many cell types and extracellular proteases (9, 14). Mechanistically, it has been suggested that physical traction resulting from interaction between ECM-associated latent TGF-β and cell-surface αV integrins ultimately leads to conformational changes of the latent complexes and to active TGF-β release (15). In addition, this conformational change is thought to allow higher accessibility of the latent complex to extracellular proteases (9). Overall physiological release of active TGF-β likely requires synergic actions of both integrins and proteases (16).

TGF-βs signal *via* a pair of transmembrane serine/threonine protein kinases known as the type I (TGF-βR1 or ALK5) and type II receptors (TGF-βR2). Binding of one of these cytokines to a TGF-βR2 dimer triggers the recruitment and subsequent transphosphorylation of TGF-βR1 which, in turn, activates the downstream canonical signaling cascade *via* the receptor-regulated SMADs (R-SMADs). BMP subfamily receptors phosphorylate SMAD1, SMAD5 and SMAD8, while TGF-β subfamily receptors mainly phosphorylate SMAD2 and SMAD3. Upon phosphorylation, R-SMADs dissociate from the receptor and form a heterotrimeric transcriptional complex with SMAD4. Typically, heterotrimers of SMAD2, SMAD3 and SMAD4 are formed, but complexes that contain homodimers of SMAD2 or SMAD3 may form as well and target distinct genes (17). Localization and concentration of SMADs are regulated by anchor for receptor activation (SARA) proteins which recruit non-phosphorylated SMADs to the activated receptors for phosphorylation. Upon receptor activation, phosphorylation of SMADs causes their dissociation from SARA and formation of SMAD2/3–SMAD4 complexes (18). Heteromeric SMAD complexes translocate and accumulate in the nucleus where they bind DNA directly but with low affinity and specificity or indirectly by interacting with other DNA-binding proteins to target specific genes for transcriptional regulation. Regulatory mechanisms are in place to avoid spontaneous transcriptional activation of TGF-β target genes. Thus, in absence of TGF-β stimuli, SKI inhibits the expression of TGF-β target genes by binding Smad Binding Elements (SBEs) complexed with SMAD4 and by recruiting other repressors and histone deacetylases (HDA) (19). SMADs turnover in the nucleus is tightly controlled. Nuclear SMAD levels are kept in check either by regulating protein turnover, before and after translocation into the nucleus, or by regulating the subcellular localization of SMADs. SMAD6 and SMAD7 are inhibitory molecules that suppress receptor and SMAD signaling functions. SMAD7 recruits SMURF2 ubiquitin ligases to TGF-β receptors to promote their ubiquitin-mediated degradation (20, 21). Inhibitory SMADs also associate with R-SMADs, preventing the formation of R-SMAD complexes with SMAD4. In addition, the expression of SMAD6 and SMAD7 induced by TGF-β family members also triggers negative feedback (22). SMAD interactions with distinct karyopherin proteins might also result in differential regulation of nuclear import depending on the SMAD, which may represent a complementary layer of regulation of the dynamics of SMAD-mediated transcription responses. Once in the nucleus, SMAD trimers interact with specific transcriptional factors as well as with regulatory proteins, to initiate transcription of specific target genes. Upon dephosphorylation by the nuclear phosphatase PPM1A, SMAD trimeric complexes disassemble and SMAD proteins are exported out of the nucleus by different exportins, such as RanBP3, thus facilitating termination of TGF-β signals (23, 24).

TGF-β receptors stimulation can also result in the activation of SMAD-independent pathways that are collectively referred to as non-canonical TGF-β signaling pathways (25). They include branches of ERK, JNK, p38 MAPK, NF-κB, PI3K-AKT, JAK-STAT and small GTPase pathways (10, 26, 27). Upon activation, direct phosphorylation of ShcA by TGF-βRI induces its association with Grb2/Sos and sequential Ras, MEK1/2 and ERK1/2 activation (28). Thus, initiation of the ERK pathway also depends on TGF-βRI mediated phosphorylation. In contrast, TGF-βRI kinase activity seems to be dispensable for the activation of other non-SMAD signaling pathways. Recruitment of the E3 ubiquitin ligases TRAF4 and TRAF6 onto the TGF-β receptors leads to intramolecular polyubiquitination of TRAF4 or TRAF6, and consequent recruitment and ubiquitination of the MAPKK kinase TAK1. This post-translational modification does not lead to TAK1 degradation, but instead to MAPK kinases (MKKs) phosphorylation by TAK1 and finally to activation of either JNK or p38 (29, 30). Eventually, TRAF6 can also interact with IKK proteins and trigger NF-κB signaling (31). In a similar way, TRAF6-mediated ubiquitination seems to be central for TGF-β-dependent activation of PI3K and AKT in the mTOR pathway (32). Finally, TGF-β can also regulate other non-SMAD pathways, including Rho-like GTPases. Yet, more work is needed to better elucidate the details of their TGF-β-dependent activation (10, 33, 34). When and how TGF-β stimulation can lead to SMAD-dependent or -independent signals is clearly cell- and context- dependent, but the intricate regulatory mechanisms that orchestrate these events remain to be fully deciphered. It has been shown that SMAD-dependent signaling is associated with clathrin-bound receptors, while non-SMAD signaling pathways originate from lipid-raft caveolar compartments (35), suggesting that the variability in signaling output might depend on functionally different receptor complexes (10). Several kinases, including some members of the MAP kinase family, can also phosphorylate R-SMADs, and thereby regulate their effector functions in a TGF-β-independent manner. As a consequence, R-SMADs activation can result from stimulation by cytokines distinct from TGF-β family members. Moreover, translocation of R-SMADs to the nucleus can induce a delayed activation of non-SMAD pathways *via* gene-expression regulation, further complicating the study of downstream molecular events (10). Overall, these multiple signaling cross-talks enable a broad diversity of responses and underlie the versatility in terms of dosage, spatiotemporal activity and cell context of this family of cytokines in regulating cellular processes. A range of inherited human diseases results from single-gene variants in several components of this tightly controlled signaling pathway. On one end of the range, these gene defects lead to exclusively developmental defects, while on the other end, they are restricted almost purely to the immune system. Therefore, comparing the phenotypic consequences of pathogenic variants in genes encoding TGF-β receptors or downstream components of the signaling machinery, and in genes encoding members of the family (TGF-β1, TGF-β2, and TGF-β3) enable to differentiate and appreciate the role of those individual cytokines in immune and non-immune cells in humans.

## Mendelian disorders affecting TGF-β signaling and presenting with immune dysregulation

The term “TGF-β signalopathies” defines a number of Mendelian disorders associated with inappropriate activation of TGF-β signaling and ultimately impacting developmental processes, immune responses, or both. A large group of these gene defects result in complex syndromes of variable expressivity encompassing a broad spectrum of cardiovascular, craniofacial, and skeletal features associated or not with impaired immune regulation (6) (**Table 1**). Those, include, among others, Loeys–Dietz syndrome (LDS) which is caused by heterozygous loss-of-function (LOF) variants in *TGFBR1* (MIM: 190181), *TGFBR2* (MIM: 190182), *TGFB2* (MIM: 190220) or *TGFB3* (MIM: 190230), *SMAD2* (MIM: 601366) or *SMAD3* (MIM: 603109) and Marfan syndrome (MFS) (MIM: 154700) caused by variants in *FBN1*, encoding fibrillin 1, the main component of extracellular matrix microfibrils that scaffolds latent TGF-β (**Figure 1**). Clinically, MFS and LDS show overlapping cardiovascular and skeletal manifestations. Yet, in contrast to LDS patients, immune regulation is not affected in Marfan patients, likely because fibrillin 1 is mainly expressed in connective and soft tissues, and muscles, while it is almost absent in sites that are exposed to environmental immune triggers such as lungs and intestine, or in sites of adaptive immune priming such as lymph nodes and spleen. Similarly, Shprintzen-Goldberg syndrome (MIM: 182212), which is caused by heterozygous variants in *SKI* features all the craniofacial, skeletal and cardiovascular manifestations of LDS but no immune dysregulation, likely due to the expression pattern of SKI. Indeed, expression of this negative regulator of the pathway, at least in vessels, seems to be regionally confined and temporally limited at early stages of development in mice (41). Contrasting with this group of diseases, the more recently identified TGF-β1 deficiency results in a prominent immune phenotype underscoring the central immunoregulatory role of this cytokine.

**Table 1:** Clinical features presenting in patients with TGF-β signalopathies.

Gene	*TGFBR1/2*	*TGFB2/3*	*SMAD2/3*	*TGFB1*	*IPO8*	*ERBIN*	*MAPK8* (JNK1)
Gene/Locus MIM number	190181; 190182	190220; 190230	601366; 603109	190180	605600	606944	601158
Phenotype MIM number	609192; 610168	614816; 615582	619656; 613795	618213	619472	/	/
Type of variant	LOF	LOF	LOF	LOF	LOF	LOF	LOF
Inheritance	AD	AD	AD	AR	AR	AD	AD
**Vascular abnormalities**	yes	yes	yes	N/A	yes	yes	no
**Ocular abnormalities**	yes	yes	yes	N/A	yes	N/A	no
**Craniofacial abnormalities**	yes	yes	yes	N/A	yes	N/A	yes
**Skeletal abnormalities**	yes	yes	yes	N/A	yes	yes	yes
**Connective tissue abnormalities**	yes	yes	yes	N/A	yes	yes	yes
**Neurological manifestations**	yes	yes	yes	yes	yes	N/A	yes
**Immunological abnormalities**							
Allergic symptoms	yes	N/A	yes	N/A	yes	yes	yes
Autoimmunity	N/A	N/A	yes	N/A	N/A	yes	N/A
Recurent infections	N/A	N/A	N/A	yes	N/A	N/A	yes
Intestinal inflammation	yes	N/A	yes	yes	yes	N/A	no
Intestinal transit problems	N/A	N/A	N/A	N/A	N/A	N/A	yes
Eosinophilic esophagitis	yes	N/A	N/A	yes	N/A	yes	N/A
Hypereosinophilia	yes	N/A	N/A	N/A	yes	yes	yes
T_H_1 cells	N/A	N/A	N/A	reduced	N/A	N/A	increased
T_H_17 cells	N/A	N/A	N/A	reduced	N/A	N/A	reduced
T_reg_ cells	increased	N/A	N/A	reduced	N/A	increased	normal
HyperIgE	yes	N/A	N/A	yes	yes	yes	N/A
HyperIgG	no	N/A	N/A	yes	yes	no	no
HyperIgM	no	N/A	N/A	no	N/A	no	no
HypoIgA	no	N/A	N/A	no	yes	yes	no
T_H_2 cytokines production	increased	N/A	N/A	N/A	N/A	increased	normal
IL-17 production	intact	N/A	N/A	reduced	N/A	intact	reduced
**TGF-β signaling**	Increased in aorta, bone, thymic tissue and CD4^+^ T cells	Increased in the aorta	Increased in the aorta	Reduce pSMAD in lamina propria mononuclear cells	Reduced pSMAD translocation in *ipo8^-/-^* zebrafish, higher pSMAD nuclear accumulation in *Ipo8^-/-^* mice aorta	Increased pSMAD nuclear accumulation in primary lymphocytes following TGF-β stimulation	Impaired SMAD-independent TGF-β signaling in fibroblasts following TGF-β stimulation
References	(36, 37)	(6, 7)	(38–40)	(2)	(3)	(4)	(5)

AD, autosomal dominant; AR, autosomal recessive; LOF, loss-of-function; N/A, not available.

### TGF-β1 deficiency causes very early onset-IBD

As mentioned above, bi-allelic LOF variants in *TGFB1* were recently identified in 3 patients from 2 unrelated families presenting with severe very early onset IBD, manifesting as bloody diarrhea with failure to thrive, and central nervous system (CNS) disease associated with epilepsy, brain atrophy and posterior leukoencephalopathy (2). Functionally, these variants affected TGF-β1 complex formation, secretion and/or bioavailability for signal transduction ultimately resulting in decreased downstream SMAD2/3 signaling. Recurrent infections and dermatitis were also reported in two patients altogether with reduced T-cell responses to stimulation with anti-CD3 and anti-CD28 as well as impaired T cell proliferation in response to diphtheria and tetanus toxoids. Immunophenotyping was performed only in one child and showed decreased memory T_reg_, T_H_1 and T_H_17 cell counts in peripheral blood, associated with reduced frequencies of T_reg_, T_H_1, T_H_17 and CD103^+^ T cells infiltrating the colon and increased serum concentration of IgG and IgE. The disease course was extremely severe as only one patient remained alive at the age of 11 years in stable condition, while in 2 others septicemia resulted in death at 25 and 39 months, respectively. Given the very severe condition of the affected children, it remains difficult to ascribe all immunological abnormalities and notably the fatal infectious complications to the lack of TGF-β1. However, the very severe IBD phenotype illustrates the key role of TGF-β1 in maintaining immune homeostasis at the intestinal barrier. Of note, *Tgfb1^-/-^* mice show no gross developmental defects but they develop immediately after birth very severe multi-organ inflammation resulting in organ failure and fatal outcome within 20-30 days (**Table 2**) (42–44). Overall data in humans and in mice converge to demonstrate the non-redundant role of TGF-β1 in maintaining immune homeostasis. In contrast to LDS, TGF-β1 deficiency is associated with severe CNS dysfunction. The mechanism(s) of this very severe neurological phenotype remain(s) not fully understood and the respective role of uncontrolled inflammation and of a developmental defect are not yet well-delineated.

**Table 2:** Mouse models for the study of immune dysregulation in TGF-β signalopathies.

Target	Mouse model	Affected cells	Immunological phenotype	References
TGF-β1	*Tgfb1^-/-^*	Systemic effects	Increased embryonic lethality; severe autoimmunity and inflammation, reduced peripheral T_reg_ cells, death around 3 weeks of age	(42–44)
	*Tgfb1^f/f^*	T cells	Wasting and severe colitis leading to lethality starting at 6 months of age, altered T cell activation, proliferation, and differentiation	(45)
TGF-βRI	*Tgfbr1^f/f^*	T cells	In Lck-Cre and CD4-Cre mice, lethal inflammation within 7 weeks of age; decreased thymic and splenic T_reg_ cells	(46, 47)
TGF-βRII	*Tgfbr2^f/f^*	T cells	In CD4-Cre mice, early-onset lethal inflammation with altered T cell development and activation	(48, 49)
SMAD2	*Smad2^-/-^*	Systemic effects	Embryo lethality before E8.5	(50, 51)
	*Smad2^f/f^*	T cells	In Lck-Cre, spontaneous low grade intestinal inflammation, increased susceptibility to experimental colitis, normal T cell development in thymus and spleen, but altered T cell activation and homeostasis	(52)
SMAD3	*Smad3^-/-^*	Systemic effects	Viable, impaired TGF-β antiproliferative effect on T cells, spontaneous chronic intestinal inflammation, and immune function defects leading to death between 1 and 8 months. In LckCre-Smad2^f/f^, severe autoimmunity and inflammation, death within 3 to 5 weeks of age	(52–54)
ERBIN	*Erbin^-/-^*	Systemic effects	Spontaneous low grade intestinal inflammation, increased susceptibility to experimental colitis	(55)
IPO8	*Ipo8^-/-^*	Systemic effects	Not explored; evidence of increased pSMADs levels in end-stage tissues	(56)
JNK1	*Jnk1^-/-^*	Systemic effects	Hyperproliferation of T cells, decreased activation-induced cell death, altered T cell subset differentiation	(57)

### Defects in TGF-β receptor 1/2 and SMAD2/3 result in Loeys-Dietz syndrome featuring atopy and intestinal inflammation

Autosomal dominant (AD) variants in genes coding for TGF-β ligands (*TGFB2*, *TGFB3*), receptors (*TGFBR1*, *TGFBR2*) or downstream effectors (*SMAD2*, *SMAD3*) (58–63) cause LDS syndrome. These gene defects share the craniofacial, cardiovascular and skeletal phenotypic core but penetrance, disease severity and immune defects are variable depending on the gene. The exact nature of the signaling defects remains difficult to appreciate. Thus, despite being inactivating when functionally tested *in vitro*, heterozygous variants in *TGFBR1* or *TGFBR2* paradoxically result in increased TGF-β signaling output, as revealed by elevated levels of phosphorylated SMAD2/3 or ERK1/2 and increased TGF-β1 levels in several tissues derived from LDS patients, including aorta, bone, and thymus (59, 61–63). Altered cell surface recycling of the mutant receptors (64), imbalance between SMAD-dependent and SMAD-independent cascades (65) or alternative ligand usage (41, 62) may explain the paradoxical increase in TGF-β signaling. Yet, more recently, data obtained in cardiovascular progenitor cell-derived smooth muscle cells (CPC-SMCs) differentiated from human induced pluripotent stem cells (hiPSC) carrying AD *TGFBRs* variants showed decreased, rather than enhanced, pSMAD2/3 and pAKT signaling following TGF-β1 stimulation (66), thus suggesting that LOF *TGFBRs* variants do indeed result in decreased pathway activation. It seems possible that epigenetic changes and compensatory mechanisms become activated overtime, thus leading to the increase in pSMAD2/3 that is observed in end-stage aortic tissues. More undifferentiated cell types might thus be better suited to interrogate the early molecular events underlying development of LDS and LDS-related disorders. Immune dysregulation with variable prevalence and severity can be observed in LDS patients. Increased risk to develop atopy including food allergies asthma, allergic rhinitis, eczema and eosinophilic gastrointestinal diseases was first reported in a cohort of *TGFBR1* or *TGFBR2* carriers (36) and confirmed by latter identification of additional cases (37). Consistent with the allergic manifestations, these patients had significantly increased eosinophil counts, elevated levels of both IgE and allergy-associated type 2 cytokines IL-5 and IL-13 which contrasted with normal non-IgE antibody isotypes. As previously reported in aortic tissues, a paradoxical increase in TGF-β signaling was observed in CD4^+^ T lymphocytes (36). Accordingly, and consistent with the role of TGF-β in driving T_reg_ cells generation (67), but at odd with the loss of tolerance, LDS patients had increased frequency of peripheral FOXP3^+^ T_reg_ cells with normal ability to suppress effector T cell proliferation (36). In addition, increased prevalence of IBD has also been reported in 2 unrelated cohorts of LDS patients (58, 68). Overall, the paradoxical enhancement of TGF-β signaling observed in LDS patients with monoallelic LOF variants in *TGFBR1* or *TGFBR2* makes it difficult to compare observations in humans with data available in mice, where complete ablation of *Tgfbr1* or *Tgfbr2* in T cells causes a rapidly fatal autoimmune disease characterized by lymphoproliferation, increased T cell activation and cytotoxic differentiation, and drastic T_reg_ cells decrease in the periphery (**Table 2**) (46–49).

In line with the role of SMAD3 downstream all 3 TGF-βs, autoimmune features and allergic manifestations have been also reported in a cohort of heterozygous *SMAD3* carriers (38). In marked contrast, the regulation of the immune response does not seem to be affected in LDS patients carrying variants in *TGFB2/3*. The lack of immune phenotype in the latter patients is in keeping with data obtained in *Tgfb2^-/-^* and *Tgfb3^-/-^* mice (69–71). Thus, these mice present perinatal mortality with multiple but little overlapping developmental defects affecting a broad range of organs, but no immune defects. Overall, these data indicate that, in humans as in mice, the three TGF-β isoforms cannot compensate each other. While TGF-β1 is key to maintain homeostasis in the immune system, TGF-β2 and TGF-β3 play complementary roles in connective tissue development.

### Aberrant regulation of TGF-β signaling due to defects in STAT3-ERBIN-SMAD2/3 complex leads to atopic predisposition

ERBB2-interacting protein (ERBIN), a member of the leucine-rich repeat and PDZ domain (LAP) family, is a SMAD-binding (72) and SARA-interacting protein (73) that has been shown to negatively regulate TGF-β signaling activation. ERBIN binds and segregates phosphorylated SMAD2/3 in the cytoplasm, preventing their translocation in the nucleus thereby dampening SMAD2/3-dependent transcription. SARA recruits non-phosphorylated SMAD2/3 to activated receptors to promote their phosphorylation and competes with SMAD2/3 for binding to ERBIN (73) (**Figure 1**). Therefore, the concentrations of these three components of the pathway and their relative binding affinities modulate the output of TGF-β signaling. Accordingly, deregulation of TGF-β signaling is observed in patients carrying heterozygous LOF variants in *ERBIN* (4). These patients present with a connective tissue syndrome including vascular abnormalities, as well as with immunological manifestations, including eczema, atopy, eosinophilic esophagitis (EoE) with hypereosinophilia and elevated IgE (4, 74). Hypertrophic cardiomyopathy observed in one patient could be recapitulated in *Erbin* deficient mice (75). Interestingly, these mice are more susceptible to experimental colitis and spontaneously develop low grade intestinal inflammation and epithelial injury over time but the role of TGF-β signaling in these manifestations was not explored (**Table 2**) (55). In primary CD4^+^ T lymphocytes from *ERBIN* patients, reduced ERBIN expression did not affect total pSMAD2/3 levels in response to TGF-β stimulation, but rather resulted in increased nuclear accumulation of pSMAD2/3, consistent with the role of ERBIN in segregating activated pSMAD2/3 in the cytoplasm. In keeping with the increased sensitivity to TGF-β conferred by *ERBIN* deficiency and with the key role of TGF-β in T_reg_ cells development, patients displayed increased counts of peripheral T_reg_ cells. Moreover, *in vitro* differentiation of iT_reg_ cells from naïve CD4^+^ T cells was increased compared to controls. Dysregulation of TGF-β signaling was associated with increased IL-4Rα expression and increased activation of the IL-4-GATA3 axis upon weak TCR stimulation of naïve *ERBIN* deficient CD4^+^ T lymphocytes. Increased *ex vivo* production of T_H_2 cytokines (IL-4, IL-5 and IL-13) by *ERBIN* deficient memory CD45RO^+^ CD4^+^ lymphocytes further suggest skewed type 2 immune responses and might account for the allergic manifestations observed in the patients. Accordingly, amelioration of the refractory allergic eosinophilic inflammation could be achieved by IL-4Rα blockade in a 16 yo *ERBIN* patient (74). Interestingly, IL-6/STAT3 and IL-11/STAT3 activation suppresses TGF-β signaling *via* formation of the STAT3-ERBIN-SMAD2/3 complex with subsequent SMAD2/3 retention in the nucleus and inhibition of the TGF-β signaling, thus highlighting the molecular intersection between JAK-STAT pathway activation and repression of TGF-β signaling (4, 76). Accordingly, enhanced SMAD signaling is reported in patients carrying dominant-negative *STAT3* variants, who show, alike ERBIN patients, elevated IgE and EoE. Overall these observations establish a link between increased TGF-β activity, impaired STAT3 activation and immune dysregulation.

### Impaired SMADs translocation due to IPO8 deficiency causes Loeys-Dietz like syndrome

Nuclear translocation to the nucleus of activated R-SMADs is a regulated process which is key for transduction of TGF-β signals. Some data have shown that the shuttling of SMADs downstream TGF-β signal depends on a member of the karyopherin family of nuclear transport receptors, importin 8 (IPO8) (77, 78). Karyopherins use RANGTP to translocate cargos through the nuclear pore complexes and once in the nucleus, they release the cargo and finally return to the cytoplasm (79). Three groups have concurrently reported biallelic LOF variants as cause of a complex syndrome overlapping with LDS, including early-onset thoracic aortic aneurysm and structural cardiac defects, joint hypermobility, skin laxity, and hernias, dysmorphic features, skeletal abnormalities, and developmental delay (3, 56, 80). As in LDS, immune dysregulation has been observed in some patients, including predisposition to allergic or intestinal inflammatory diseases, hyperIgE, hyperIgG, hypoIgA, and hypereosinophilia (3). Evidence in zebrafish and in mouse (**Table 2**), indicate that *Ipo8* deficiency can disrupt TGF-β and or BMP signaling and thereby explain the vascular, skeletal and immunological anomalies observed in the patients. Yet, whether IPO8 loss results in increased or decreased pSMADs shuttling with consequent increased or decreased TGF-β pathway activation is still unclear. Immunohistochemistry analysis of ascending aortic sections in *Ipo8^-/-^* mice revealed increased nuclear pSMAD2/3 as compared to WT animals. Along with the observation of decreased expression of TGF-β negative regulator Smad7, this might suggest increased pathway activation (56). In contrast, decreased pSMAD1/5/9 nuclear accumulation was found in *ipo8^-/-^* zebrafish embryos. Accordingly, expression of transcripts encoding *smad7* and other TGF-β dependent genes were reduced in 13 hpf and 24 hpf zebrafish embryos, pointing to decreased - rather than increased - pathway activation in this model (3). Taken together, defective SMAD translocation might be considered as an early event underlying disease development, as it can be observed in undifferentiated cell types, while compensatory mechanisms acting overtime might lead to increased SMADs phosphorylation in end-stage tissues. Of note, the development of thoracic aortic aneurysms development is recapitulated in *Ipo8* deficient mice but their immune system has not been studied (56). Further investigation will be necessary to define whether and how defective IPO8-dependent nuclear translocation may underlie immune dysregulation in *IPO8* deficient patients.

### Impaired JNK1-dependent MAPK signaling pathway cause chronic mucocutaneous candidiasis and connective tissue disorder

A heterozygous LOF variant in *MAPK8*, the gene encoding the c-Jun N-terminal kinase 1 (JNK1) was recently reported in a three-generation kindred with a complex syndrome combining chronic mucocutaneous candidiasis and a connective tissue disorder but no evidence of aortic aneurysm or dissection (5). JNK1 is one of the three kinases of the JNK family and a member of the mitogen-activated protein kinase (MAPK) pathway (81). As shown in **Figure 1**, TGF-β can induce the activation of MAPK kinases (specifically MKK4 and MKK7) resulting in JNK1 phosphorylation. In turn, JNK1 phosphorylates activator protein-1 (AP-1) transcription factors, including c-Jun and ATF-2, and ultimately triggers SMAD2/3-independent transcription (82). Accordingly, fibroblasts derived from patients carrying heterozygous *MAPK8* LOF variants showed impaired c-Jun/ATF-2 signaling in response to TGFβ but normal ERK1/2, p38, and SMAD2/3 activation. Dysregulation of TGF-β-dependent signals was suggested to account for the connective tissue disorders. Of note, patients also displayed higher proportions of T_H_1 cells and lower proportions of T_H_17 cells than controls, while frequencies of T_H_2 and T_reg_ cells were normal. In addition, naïve CD4^+^ T cells from patients carrying the heterozygous *MAPK8* variant produced less IL-17A and IL-17F in T_H_17-polarizing conditions *in vitro*. Overall these results are consistent with the role played by JNK1 in T_H_17-cell activation and differentiation in mice (**Table 2**) (57, 83) and with TGF-β requirement for T_H_17 differentiation in both mice (84) and humans (85–87) and suggest that impaired T_H_17 development underlies the predisposition to chronic mucocutaneous candidiasis.

## Conclusions

Patients carrying single gene defects in individual components of the TGF-β signaling machinery are invaluable resources to dissect the relative contribution, signaling cross-talks, specificity and redundancy of each of those components in the orchestration of the immune response. Human TGF-β1 deficiency results in very early onset severe IBD, demonstrating the non-redundant role of TGF-β1 in suppressing intestinal inflammation and supporting the hypothesis that the aberrant TGF-β1/SMAD signaling observed in active lesions of IBD patients participate in disease pathogenesis (88). Increased Smad7 associated with downregulation of the TGF-β signaling, shown to amplify gut inflammatory responses in those patients (89), could be targeted by specific antisense oligonucleotides (mongersen), which however provided conflicting results in phase II/III studies (89–91). Pathogenic monoallelic variants in *TGFBR1/2* as well as in *SMAD3* predispose to IBD, strengthening the key role of the SMAD3 canonical pathway for maintaining intestinal homeostasis, a role previously supported by GWAS studies in common multifactorial forms of IBD (1) as well as by Smad3 inactivation in mice (53, 54). Strikingly, these gene defects also predispose to allergy, overall suggesting that TGF-β is instrumental in humans to control immune responses to environmental triggers. While TGF-β1 deficiency may benefit from supplementation by this cytokine, the complexity of TGF-β signaling remains a major challenge to define appropriate therapeutic strategies in other defects impairing either chains of the receptor or downstream elements of the signaling cascade.

## Author contributions

MR, NC-B and MP wrote the manuscript. MR prepared the tables and the figure with BioRender. All authors contributed to the article and approved the submitted version.
